# Effect of Environmental Factors on Bone Remodelling: A Narrative Review

**DOI:** 10.7759/cureus.96490

**Published:** 2025-11-10

**Authors:** Bawan Hama, Shahan Hama, Abdulrahman Kashkosh, Fadel Jesry, Isbah Munir, Maha Ejaz, Hassan Kazemi

**Affiliations:** 1 Trauma and Orthopaedics, King's College Hospital NHS Foundation Trust, London, GBR; 2 Trauma and Orthopaedics, Royal Devon University Healthcare NHS Foundation Trust, Exeter, GBR; 3 Trauma and Orthopaedics, Sheffield Teaching Hospitals NHS Foundation Trust, Sheffield, GBR; 4 Trauma and Orthopaedics, Mid Yorkshire Teaching NHS Trust, Wakefield, GBR; 5 Trauma and Orthopaedics, Bradford Teaching Hospitals NHS Foundation Trust, Bradford, GBR; 6 Plastic Surgery, Bradford Teaching Hospitals NHS Foundation Trust, Bradford, GBR; 7 Trauma and Orthopaedics, Bolton NHS Foundation Trust, Bolton, GBR

**Keywords:** bisphosphonate use, bone health, bone remodelling, environmental factors, osteoblast, osteoclast, osteoporosis, physical activity, smoking

## Abstract

Bone remodelling is a finely balanced process primarily maintained by two fundamental cell types: osteoblasts and osteoclasts. Many intrinsic and extrinsic factors can affect this process leading to pathological conditions such as osteoporosis. This narrative review focuses on some of the key environmental factors affecting bone remodelling including physical activity, lifestyle factors, nutrition, pollution, medications, and environmental stressors. We will attempt to identify some of the key pathways affected, and how this impacts bone health, whilst using this knowledge to look at possible therapeutic options.

## Introduction and background

The living skeleton is a dynamic organ constantly undergoing resorption and formation [1]. This fine balance, known as bone remodelling, maintains healthy mineralised bone, which can support the weight of the body. Bone tissue develops from mesenchymal stem cells (MSCs), which can differentiate into chondrocytes or cells of the osteoblast lineage (including osteoblast progenitor cells, osteoblasts, and osteocytes). Bone homeostasis is maintained by a fine balance between osteoblasts (bone-forming cells) and osteoclasts (bone-resorbing cells), derived from bone marrow monocytes [2,3].

Environmental and genetic factors affecting bone health and remodelling have long been documented in the literature. With a growing population, improved diagnostic methods, and a greater understanding of biochemical pathways, it is important to ascertain how largely modifiable factors affect bone health. This narrative review, therefore, examines the impact of environmental factors on bone remodelling, with particular emphasis on lifestyle factors, pollutants, nutrition, pharmacological agents, and environmental stressors. By integrating findings from experimental and clinical studies, we aim to elucidate the mechanisms by which these exposures influence skeletal health and to identify potential avenues for therapeutic modulation.

Bone remodelling

**Figure 1:**
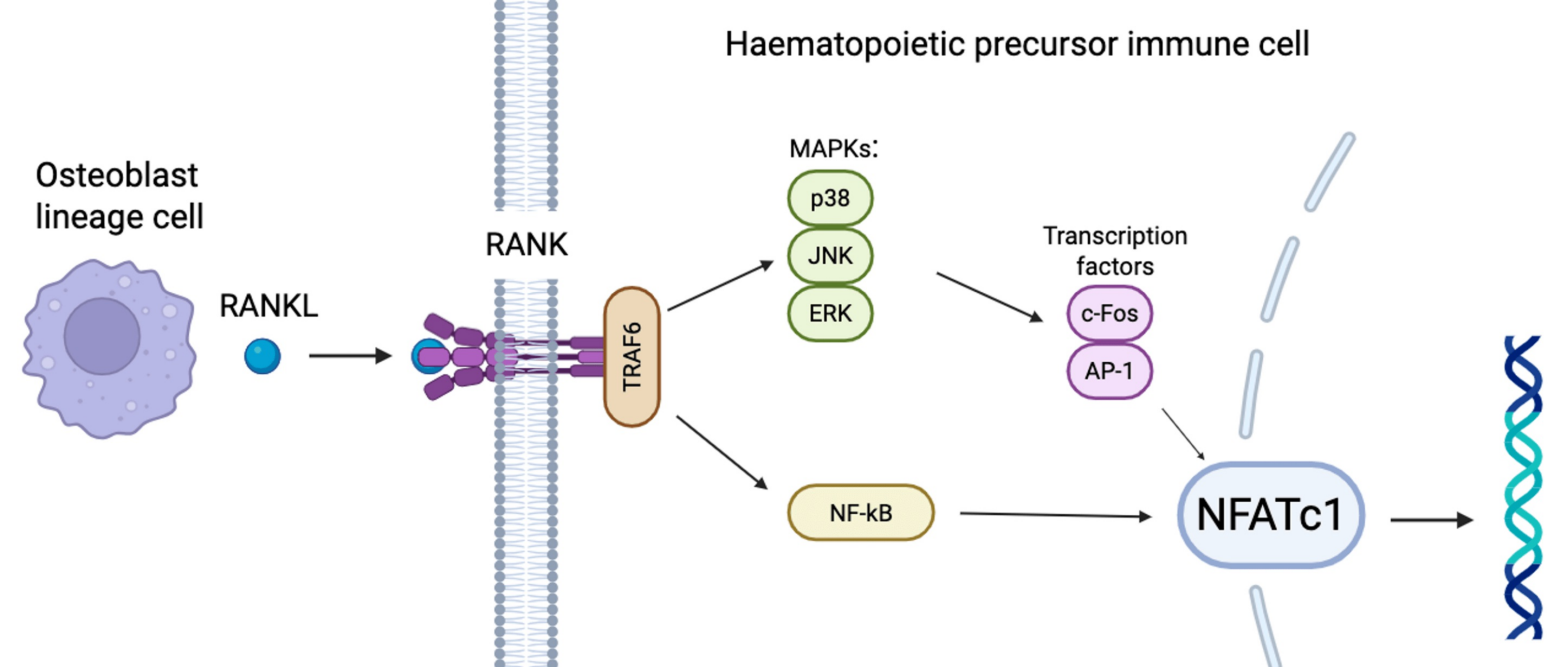
RANKL/RANK signalling pathway driving osteoclastogenesis RANKL secreted by osteoblast lineage cells binds to the RANK receptor on haematopoietic precursor immune cells (monocytes and macrophages), leading to recruitment of TRAFs (particularly TRAF6). This activates MAPK pathways (p38, JNK, ERK) and NF-κB signalling. MAPKs induce transcription factors c-Fos and AP-1, which cooperate with NF-κB to upregulate NFATc1, the master regulator of osteoclast differentiation. RANKL: receptor activator of nuclear factor κB ligand; RANK: receptor activator of nuclear factor κ B; TRAF: tumour necrosis factor receptor-associated factors; MAPK: mitogen-activated protein kinase; NFATc1: nuclear factor of activated T cells 1 Image Credit: Authors; created with BioRender.com

Bone remodelling is primarily controlled by the interplay between three essential molecules: receptor activator of nuclear factor κB ligand (RANKL), RANK, and osteoprotegerin (OPG) [4]. RANK is a member of the tumour necrosis factor receptor (TNFR) superfamily. Alone, RANK lacks the enzymatic activity needed to activate downstream signalling molecules [5]. Instead, it requires activation via binding of RANKL. RANKL is produced by osteoblast lineage cells. Once it binds to its receptor, RANKL initiates a series of intracellular signals by directly recruiting adaptor molecules such as TNFR-associated factors (TRAFs), which in turn activate mitogen-activated protein kinases (MAPKs) and nuclear factor-κB (NF-κB) [6]. RANK's effect on these downstream molecules is precisely and sequentially controlled. It eventually leads to the commitment of haematopoietic precursor immune cells (monocytes and macrophages) to the osteoclast lineage (Figure 1) [7]. Activated osteoclasts release proteolytic enzymes that destroy connective tissues in bones, whilst secreted acids resolve the mineral part of bones [8]. Animal studies have shown that eliminating RANKL and RANK inhibits bone loss and osteoporosis [9].

**Figure 2:**
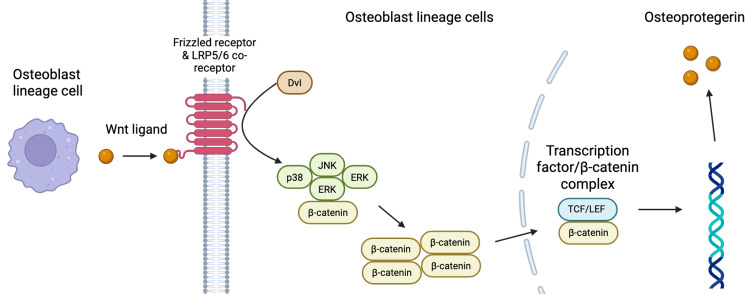
Canonical Wnt/β-catenin signalling in osteoblast lineage cells promoting OPG formation Wnt ligands released by osteoblast lineage cells bind to Frizzled receptors and LRP5/6 co-receptors on neighbouring osteoblast lineage cells. This interaction recruits Dishevelled (Dvl), inhibiting β-catenin degradation and allowing β-catenin to accumulate and translocate into the nucleus. Here, β-catenin forms a complex with transcription factors (TCF/LEF), activating expression of genes to form OPG. OPG: osteoprotegerin Image Credit: Authors; created with BioRender.com

One mechanism available to modulate osteoclast conversion is OPG. Like RANKL, OPG is secreted by the osteoblast lineage cells. It acts as a decoy ligand for RANK, binding to RANKL and preventing osteoclastogenesis [8]. Probably the most important determinant in bone resorption is the RANKL/OPG ratio. This ratio is controlled by numerous endogenous cytokines, hormones, and transcription factors [10]. One pathway by which this ratio is controlled is via the canonical Wnt/β-catenin signalling pathway [11]. When Wnt ligands (Wnt 3a and 5a) bind with their receptor complexes, the Wnt/β-catenin pathway is activated. This leads to the accumulation of β-catenin in the osteoblastic cytoplasm. The β-catenin regulates osteogenic gene transcription in the nucleus (including OPG and RUNX2) and the resultant OPG binds with RANKL to inhibit osteoclastogenesis (Figure 2) [12].

Osteoporosis

Osteoporosis (OP) is a chronic metabolic bone disorder affecting 200 million people globally and accounting for nine million fractures [13]. It is characterised by an imbalance between osteoblast and osteoclast function, and often remains asymptomatic until a fracture occurs. The WHO bases a diagnosis of OP on a bone mineral density (BMD) measurement with a T-score ≤ 2.5 standard deviation values below the mean peak of a young healthy adult [14]. OP is often classified into primary subtypes (postmenopausal and senile), and secondary forms (such as diabetic and glucocorticoid-induced) [15]. OP initiation is associated with apoptosis of osteoblasts and increased osteoclast proliferation, leading to bone loss and subsequent reduced BMD. Any abnormality in the bone matrix microenvironment can give rise to OP [16].

The primary causative factors affecting OP include oestrogen deficiency, oxidative stress, and inflammation. As humans age, fewer osteoblasts are generated from progenitor cells in favour of adipocytes. The exact mechanism driving this shift remains largely unknown, although the Notch signalling pathway is known to play a critical role in progenitor cell fate [15]. In immature osteoblasts, Notch activation suppresses their differentiation, leading to osteopenia. Conversely, in osteocytes, increased Notch signalling (via NICD1) induces OPG production, reducing bone resorption. In the myeloid lineage, inhibition of Notch signalling decreases osteoclast activity [17].

Different bone cells express distinct Notch receptors and ligands, which helps us understand these varied effects. In a murine model of Notch2-related skeletal disease, an antibody against the Negative Regulatory Region of Notch2 reversed OP [18]. Whilst promising, translating this to humans remains challenging because systemic Notch inhibition can cause gastrointestinal, haematopoietic, and vascular side effects [15].

## Review

Methods

Databases including PubMed, Embase, Cochrane Library, and Google Scholar were searched using the following keywords: bone, remodelling, environmental factors, physical activity, smoking, alcohol, obesity, pollution, pharmacological, nutrition, vitamin D, calcium, and stress.

Results

Physical Activity

Mechanical stimulation regulates the process of bone development, repair, and regeneration by influencing different cell types. Osteocytes embedded within the bone matrix are connected via dendritic cells through the lacunocanalicular network (LCN) [19]. These osteocytes in the LCN are capable of converting mechanical stimuli into intracellular signals integral to bone health. These signals regulate the functions of osteoblasts and osteoclasts to upregulate bone turnover and bone deposition [20]. Mechanosensing by osteocytes (and osteoblasts) detects mechanical loading via several types of cell-surface receptors (integrins, connexins, purinergic receptors, Piezo1/2, focal adhesions) [19].

The Piezo family of proteins (Piezo 1/ 2) has been specifically indicated in bone development and homeostasis [21]. Piezo proteins are the largest plasma membrane ion channels identified in the human body thus far [22]. These are nonselective mechanical cationic channels. Under mechanical stimuli, Piezo channels are opened, allowing cations to cross the membrane, culminating in cellular mechanotransduction leading to adaptation to the microenvironment [23]. Rapid influx of calcium through Piezo channels activates a whole host of downstream signalling pathways, including calcineurin, CAMK, Akt, ERK, β-catenin, and PI3K. Through gene expression (such as RUNX2 and RANKL/OPG), the end product is to favour increased osteoblast differentiation and activity, driving MSCs towards the osteoblast lineage, and inhibiting osteoclastogenesis via altering of RANKL/OPG ratio [24].

Physical activity also works indirectly on bone health by reducing inflammation. Exercise upregulates anti-inflammatory factors and attenuates proinflammatory factors. Studies have suggested that pro-inflammatory cytokines (such as TNF-α and IL-6) were reduced through running, and bone formation was promoted by weight-bearing exercise [25].

Lifestyle Factors

Smoking: During smoking, there is incomplete combustion of the tobacco products, leading to the generation of many toxic chemicals. Those incriminated in bone healing primarily include the polycyclic aromatic hydrocarbons (PAH): benzopyrene and 7,12-dimethylbenzanthracene [26]. Prolonged exposure to PAHs may result in OP, via an imbalance in bone homeostasis progressing to joint inflammation and bone deterioration. PAHs suppress bone synthesis by osteoblasts and bone resorption by osteoclasts [27]. One pathway this has been theorised is via the PAH led formation of aryl hydrocarbon receptor (AhR) ligands. PAHs are potent activators of AhR, pushing them into an overactive state. AHRs are transcription factor proteins that, when activated, cause accelerated osteoclast development [28]. Activated AhR acts as a co-regulator alongside RANKL, enhancing osteoclast differentiation and bone resorption. Furthermore, AhR has been demonstrated to control mitochondrial biogenesis during osteoclast development, and smoking-induced overactivation could dysregulate energy supply in osteoclasts, leading to inefficient and uncoordinated remodelling [28]. In this way, PAH from tobacco smoke can overactivate AhR and induce bone resorption. Further studies have demonstrated that smoking significantly reduces OPG levels via modulation of the canonical Wnt/β-catenin signalling pathway, leading to an increased RANKL/OPG ratio and upregulating osteoclast function [11].

Indirectly, cigarette smoking has been shown to promote vasoconstriction and affect angiogenesis. This is primarily induced through the effects of nicotine, which stimulate nicotinic receptors in the autonomic nervous system, affecting a sympathetic drive to facilitate catecholamine release from postganglionic nerve endings. This inevitably leads to vasoconstriction and impaired tissue perfusion [29]. Smoking also appears to impair angiogenesis, most evident in early fracture healing, whereby smokers exhibit reduced levels of angiogenic markers such as vascular endothelial growth factor (VEGF) and von Willebrand factor (vWF) with resultant decrease in microvessel formation [30]. This suggests smoking impairs fracture haematoma formation.

Alcohol: Although the negative effects of alcohol are widely known, a Lancet study noted its global consumption has increased by 10% from 1990 to 2017 [31]. With regard to the skeletal system, multiple pathways are altered with heavy or chronic alcohol consumption. Rat studies have shown disturbed vitamin D metabolism [32], whilst a pathway identified in its effects on bone health is the direct modulation of gene expression by alcohol, culminating in excess RANKL expression by osteoblasts, inducing osteoclastogenesis [33].

An important intracellular molecular signalling pathway involved in bone healing is the Forkhead box O (FoxO) family of transcription factors. FoxOs promote osteogenesis via the differentiation of MSCs [34]. These cells then differentiate into osteoblasts and chondrocytes to form a callus. Overactivation of the FoxO signalling pathway in the presence of excess alcohol disrupts this callus formation and impairs bone healing [35]. This occurs because alcohol increases reactive oxygen species in exposed cells. This oxidative stress overactivates FoxO proteins, which suppresses canonical Wnt signalling, a vital pathway for MSC differentiation [36].

Other important cytokines involved in bone healing include TNF-α and IL-1β, which are also affected by alcohol intake and can affect bone healing. In the early inflammatory phase of fracture healing, these cytokines are recruited to help initiate inflammation as part of the normal physiological response. TNF-α and IL-1β have long been shown to have a negative effect on bone healing [37,38]. Perrien et al. demonstrated in rat models that chronic alcohol consumption significantly elevated levels of both TNF-α and IL-1β in bone marrow [39]. This study showed impaired new bone formation and directly linked elevated levels of TNF-α to induced pathologies such as osteopenia and OP.

Obesity: Obesity can be defined as excessive fat accumulation that can lead to impaired health. The WHO defines obesity as a body mass index (BMI) of 30 kg/m^2^ or above [40]. The detrimental effect of obesity on a host of different body systems is well documented in the literature; however, the relationship between obesity, bone health, and fractures is less straightforward. In the 1990s and early 2000s, studies suggested a positive relationship between BMI and BMD [41,42]. More recently, studies in postmenopausal women suggest that as BMI increases, so does the risk of humeral fractures, whereas the risk of hip fractures reduces [43]. In light of these conflicting findings, various mechanisms and pathways have been investigated to show a beneficial or detrimental relationship between obesity and bone health.

One such mechanism includes the increased mechanical loading with obesity triggering maintenance of bone mass. Moreover, obese patients have greater subcutaneous tissue, especially around the pelvic region, and this could help “cushion” a fall and reduce the incidence of hip fractures [41]. Obesity also favours increased aromatization of androgens to oestrogens in subcutaneous adipose tissue. Oestrogen has a positive effect on bone mass and mineralisation, and its stark reduction in post-menopausal women is a major cause for OP. Via the presence of additional oestrogen, osteoclast differentiation is inhibited through oestrogen binding to RANKL [44].

Alongside its role in sex hormone synthesis, adipose tissue also secretes adipokines and cytokines crucial in bone metabolism. The primary adipokines affecting bone are adiponectin and leptin. Adiponectin receptors are present on both osteoblasts and osteoclasts, and it is able to induce RANKL and inhibit OPG production to directly increase osteoclastogenesis [45]. However, whilst this relationship is the case in postmenopausal women, the same is not replicated in premenopausal women, indicating a confounding factor of sex hormones on the effects of adiponectin [41]. Leptin, in contrast, appears to impact bone health both directly and indirectly. Leptin receptors are present on osteoblasts and chondrocytes, and their activation may impact bone health through fibroblast growth factor 23 (FGF-23). Mice studies show hypoleptinaemia (even in obese mice) predisposes to low BMD and mineralisation [46]. Leptin also acts indirectly, through its central effects on the ventromedial hypothalamus to stimulate noradrenergic signalling at osteoblasts, and by suppressing serotonin synthesis and receptor activity, thereby removing serotonin’s inhibitory effects on bone growth [47,48].

Pollution

Environmental pollutants pose a significant risk to health due to their effect on multiple biological systems and pathways. Studies have established a connection between pollution and bone disorders primarily due to low-grade inflammation, endocrine dysfunction, and oxidative stress [49]. Fine particulate matter (PM) entering the bloodstream via inhalation is known to trigger the release of proinflammatory cytokines [50]. Studies have shown that PM 2.5 μm induces elevated levels of proinflammatory monocytes and T cells [51]. PM between 2.5 μm and 10 μm induces the release of RANKL [52], and subsequent survival and maturation of osteoclasts and promoting bone resorption [53]. Furthermore, exposure to biomass fuel-derived pollutants decreases levels of OPG, further favouring uninhibited RANKL activity and skewing the RANKL/OPG ratio [52] and reinforcing the negative relationship between pollution and bone remodelling.

Other mechanisms by which pollutants induce inflammation are via the aryl hydrocarbon receptor (AhR) signalling pathways [54]. Alongside its role described under the “smoking” subheading, AhR is crucial in regulating both effector and regulatory T cells [55]. AhR has been shown to shift the balance of T cells from a regulatory to pro-inflammatory state, further affecting bone remodelling. Studies have also shown that activation of this pathway affects osteogenic differentiation, inhibiting differentiation of bone marrow stem cells into osteoblasts [56].

Nutrition

Calcium: Calcium is a crucial element integral to bone mineralisation. Of the body’s total calcium, 99% is stored in bone. Here it exists in the form of hydroxyapatite, responsible for bone mineralisation [57]. Randomised controlled trials have confirmed a relationship between the levels of calcium consumption and BMD [58]. Calcium deficiency is a main risk factor for OP. Calcium resorption from bone, secondary to low serum calcium levels, is a primary way in which OP occurs. This reduction in calcium levels arises from reduced gut absorption (mainly seen in post-menopausal women) [59]. This association between waning calcium levels and increasing incidence of OP has been primarily attributed to reduced levels of oestrogen in postmenopausal women [60]. Studies have shown normalisation of duodenal calcium absorption after administration of 17β-estradiol replacement therapy [61]. This works by upregulating the expression and function of plasma membrane calcium pump (PMCA1b), a key protein involved in duodenal absorption of calcium. The expression of this protein has been shown to be significantly reduced in postmenopausal women as compared to premenopausal women.

Vitamin D: Calcitriol, the active form of vitamin D, is an essential hormone responsible for maintaining normal bone homeostasis. Its production starts as vitamin D3, provided in the normal diet and from its production in the skin after exposure to sunlight. Vitamin D3 is hydroxylated in the liver to produce 25-hydroxyvitamin D3, which is then hydroxylated again into 1,25-dihydroxyvitamin D3 (calcitriol) in the kidneys [62]. Hypovitaminosis D is a known risk factor for OP in adults. Outila et al. demonstrated that a majority (61%) of Finnish adolescents were deficient in vitamin D during winter months; in these patients, the hypovitaminosis D was associated with a low forearm BMD [63].

Vitamin D has two main functions. Firstly, its primary function is to increase gastrointestinal absorption of calcium. It works by binding to the vitamin D receptor (VDR), a nuclear hormone receptor. This VDR is present throughout the body, including in the duodenum and kidneys. Activation of VDR by vitamin D causes increased absorption of calcium and contributes to normal bone remodelling [62]. This calcium is crucial for bone mineralisation. Low vitamin D levels contribute to reduced GI absorption of calcium, resulting in a compensatory increase in parathyroid hormone (PTH). PTH acts to increase serum calcium levels by strongly stimulating osteoclast activity [64].

Vitamin D’s secondary function works more locally to promote osteoclast activity and bone resorption. It works by once again binding to the VDR on progenitor cells. Activation of VDR results in transcription of target genes, including RANKL expression in osteoblasts. As previously explored, RANKL interaction with its receptor (RANK) stimulates increased serum calcium levels via osteoclastogenesis and bone resorption [65].

Protein: Protein constitutes half of the volume of bone and a third of its mass [66]. About 90% of this bone protein is Type I collagen. Type I collagen forms triple-helical fibrils, which are packed into bundles of strong fibers [67]. These fibers act as a framework to allow mineralisation of bone. Amino acids from protein are required for the production of intra- and extracellular bone proteins. Furthermore, amino acids can influence calcium-phosphate homeostasis. Dietary proteins stimulate the production of insulin-like growth factor-1 (IGF-1) from hepatic cells. IGF-1 has a dual function: enhancing calcitriol production in the kidneys to increase intestinal absorption of calcium and phosphate, and increasing renal reabsorption of phosphate [68]. Amino acids such as arginine and lysine also act directly at the GI lumen to promote calcium translocation from the luminal to contraluminal side of the mucosa [69]. By acting to both increase IGF-1 and directly influence calcium absorption, a diet with sufficient protein positively influences bone mineralisation.

Malabsorption: Malabsorption, secondary to a number of aetiologies including gastrointestinal disease, bariatric surgery, and anorexia nervosa, can lead to reduced absorption of vitamin D and calcium, resulting in negative skeletal consequences [70]. The most prevalent global malabsorption condition is coeliac disease, affecting 0.7% of the world’s population. In longstanding coeliac disease, characterised by intestinal villus atrophy, crypt hyperplasia and lymphocytic infiltration, low BMD is a common finding resulting from secondary hyperparathyroidism elicited by malabsorption of calcium and vitamin D [70,71]. Several studies have shown an improvement in BMD following adherence to a gluten-free diet, with improvements in vitamin D levels as well as parathyroid levels [72-74]. Vitamin D is thought to have a bidirectional relationship with malabsorptive conditions such that poor absorption of vitamin D may itself lead to worsening disease status due to its extraskeletal immunomodulatory and metabolic effects [75]. Vitamin D plays a key role in immune modulation. It can act on innate immunity by inhibiting LPS-induced p38 activation and IL-6 and TNF-α production by monocytes and downregulates TLR-9 expression, whilst in adaptive immunity, vitamin D inhibits and downregulates Th1/Th17, whilst upregulating Treg and Th2 [70,76]. Vitamin D is also involved in the production of anti-inflammatory cytokines (such as IL-4 and IL-10). Poor absorption of calcium and vitamin D is the primary mechanism by which malabsorptive conditions lead to low BMD and inadequate bone mineralisation. 

Medication

Polypharmacy is a growing consequence of the modern world. With the advancement of medicine and as people live longer, the availability of medications to treat conditions has grown. This section will look at some of the common medications widely used and how (as a side effect) they can interfere with bone remodelling. We will not focus on medications aimed specifically at preserving bone health (such as antiresorptive and anabolic drugs).

Glucocorticoids (GCs): GCs are widely used in the treatment of various inflammatory conditions. In the United Kingdom, 1% of the population are taking oral GCs, and this figure rises to 3% in the elderly [77]. A known side effect is its harmful effects on bone remodelling, predisposing to increased fracture risk secondary to GC-induced OP. This risk of fracture from systemic GCs is dose-dependent, and the most likely site of fracture is the spine, followed by the ribs and pelvis [1,78]. It is also important to distinguish between the physiological and therapeutic roles of GC in the skeleton. Physiological concentrations of GC are essential for appropriate differentiation of stromal progenitor cells towards osteoblast lineage rather than adipocytes, crucial in bone formation and accumulation of appropriate BMD [79]. Whilst the exact mechanisms of GCs' effects on bone are complex, their effects generally act via the glucocorticoid receptor (GR), expressed widely in all skeletal cell types. The GR is a transcription factor located in the cytoplasm, which once activated by its GC ligand translocates to the nucleus where it can bind directly to target genes, or interfere with other transcription factors [79,80].

At therapeutic levels, GC induces diversion of progenitor cells away from osteoblast lineage towards adipocyte lineage. This ultimately limits the number of functional osteoblasts forming, with resultant increase in bone marrow adiposity [79,81]. Studies on bone marrow stromal progenitor cells from GC-treated rodents demonstrated reduced osteoblastogenesis and enhanced adipogenesis [82]. These progenitor cells appear to show decreased expression of osteogenic transcription factors such as runt-related transcription factor 2 (RUNX2) and simultaneously increased expression of adipogenic transcription factors [79]. However, whilst this contributes to long-term bone loss, the initial phase of bone loss is mediated by the rapid induction of osteoclasts. By directly inhibiting osteoblast differentiation and inducing osteoclasts, GCs are a major cause of secondary OP.

Methotrexate (MTX):* *MTX is an antimetabolite used in several rheumatological and oncological conditions. MTX works by preventing DNA synthesis and repair during replication. MTX inhibits dihydrofolate reductase activity, leading to the cessation of purine nucleotides and thymidylates synthesis, essential for nucleic acid synthesis and repair, and cell replication [4]. Low-dose methotrexate (<25 mg/week) is indicated in rheumatological conditions, and high-dose methotrexate (HDMTX) (100-1000 mg/m^2^) is indicated in oncological conditions [83] and is the most used antimetabolite in the treatment of childhood cancers, particularly acute lymphoblastic leukaemia (ALL). HDMTX has been shown to have significant adverse effects on bone growth in children [84].

Clinical studies show that HDMTX in combination with corticosteroids results in depressed bone formation, enhanced bone resorption, and longitudinal growth arrest [85]. Since ALL treatment often involves multiple drug therapies, there is sparse literature on the effect of HDMTX alone on bone remodelling in human subjects, and many of the relevant studies focus on animal models. Studies suggest HDMTX causes increased thickness in the hypertrophic zone of the growth plate [86]. Other studies have demonstrated reduced chondrocyte proliferation, decreased collagen-II production, and increased chondrocyte apoptosis (via the Fas/FasL death receptor pathway) [87]. Thinning of newly formed primary spongiosa was observed in the adjacent metaphyseal bone (reflecting the thinning growth plate) [85].

Low-dose MTX, used as a disease-modifying anti-rheumatic drug (DMARD) in the treatment of rheumatoid arthritis, slows down articular damage in active disease [85]. A primary mode of action of MTX is its ability to inhibit inflammation via increased extracellular adenosine concentration. Fragility fractures have been reported in adults on long-term, low-dose MTX [88]; however, most clinical studies and cohort analyses do not show a clear, independent detrimental effect of low-dose MTX on BMD once confounding factors (disease activity, concurrent GC use, age, menopause, BMI) are accounted for [89,90].

Aromatase inhibitors (AIs): AIs are a group of drugs used in the treatment of breast cancer, primarily in postmenopausal women. AIs include anastrozole, letrozole, and exemestane. They work by blocking the P450 cytochrome enzyme CYP19A1 (aromatase), which converts androgens to oestrogen [91]. In postmenopausal women, the ovaries are no longer the primary producers of oestrogen, and instead, it is the conversion of androgens that drives most of the oestrogen production. By blocking this pathway, AIs work to reduce oestrogen levels and are utilised extensively in oestrogen receptor-positive breast cancers. In postmenopausal women, who are already at an increased risk of OP and fractures, the effects of AIs on bone health are an important consideration.

It is first important to understand the role of oestrogen in bone remodelling. Oestrogen regulates bone resorption by directly inhibiting RANKL production, preventing its binding to the RANK receptor on osteoblastic precursors [92]. Oestrogen is thought to also directly regulate the production of bone resorbing cytokines, progenitor cell differentiation, and inhibition of apoptosis in osteoblasts and osteocytes [93]. In concert, the function of oestrogen favours dampening bone turnover.

Studies have shown that bone loss at the beginning of menopause progresses at a rate of 2% a year for the first years, followed by 1% for the decades after. In post-menopausal women on AIs, this rises to 2.6%. Interestingly, in pre-menopausal women on both AIs and gonadotropin-releasing hormone (GnRH) agonists (to achieve complete ovarian suppression), the rate of bone loss approaches 7% [94].

By blocking oestrogen, AIs increase bone turnover, reduce BMD, and increase fracture risk. The Arimidex, Tamoxifen, Alone or in Combination (ATAC) trial was a significant trial in the field of breast cancer [95]. In the five-year multicentre, double-blind, randomised control trial, ATAC demonstrated that anastrozole was superior to tamoxifen in postmenopausal women with hormone receptor-positive early breast cancer, with improved disease-free survival and a lower incidence of contralateral breast cancer [95]. However, when assessing bone health outcomes, the trial demonstrated that there was a significantly higher incidence of fracture with anastrozole (7.1%) compared with tamoxifen (4.4%) (P < .0001) at the 40-month safety follow-up [94,95].

Stress

Chronic psychological stress is associated with dysfunction of the endocrine system, particularly the hypothalamus-pituitary-adrenal (HPA) axis [96]. Studies have indicated that during stressful periods, cortisol levels increase approximately nine times as compared with levels in a relaxed state [97]. Cortisol upregulates RANKL and downregulates OPG expression in osteoblasts, affecting osteoclast differentiation and activation, and favouring bone resorption [98,99]. In murine cell studies, cortisol dose-dependently impairs osteoclastogenesis and osteoblastogenesis, and concurrently increases osteoblast and osteocyte apoptosis [100].

Furthermore, stress causes hyperactivation of the sympathetic nervous system. Osteoblasts and osteoclasts possess receptors for neuropeptides and noradrenaline, suggesting a role for these signalling molecules in bone remodelling [101]. β2-adrenergic receptors (β2ARs) present on osteoblasts act as the functional receptors for norepinephrine, the primary sympathetic neurotransmitter. Activation of the β2-ARs increases bone resorption through modulation of RANKL and OPG produced by osteoblasts or stromal cells, leading to activation of osteoclasts [101,102]. Overactivation of the sympathetic nervous system appears to predispose to increased bone resorption.

Discussion

Bone health and remodelling are complex processes involving numerous pathways, working in synergy to maintain normal bone homeostasis. Factors affecting this equilibrium can alter bone health, and in the modern world, it is important to understand the role of environmental factors in this equation. The environmental effects on these pathways clearly play a significant role in bone remodelling. Understanding the effects allows for more targeted therapeutic options. Recommendations for changes in lifestyle are thus often finely balanced to ensure a holistic approach.

A review of the literature by the National Centre for Smoking Cessation and Training (NCSCT) showed that the risk of fractures in smokers is higher than in ex-smokers, and in these patients, the risk was higher than in non-smokers. Their study also indicated that the effect of smoking on BMD is partially reversible after stopping smoking, whilst also charting a dose-dependent correlation between pack years smoked and fracture risk [103]. More modern alternatives, such as e-cigarettes, have been touted as a safer alternative. Public Health England (PHE) guidance suggests it is 95% safer than traditional cigarettes [104]. However, while e-cigarettes contain fewer toxins, recent rat studies suggest chronic exposure induces microfractures without affecting cortical bone strength [105]. Even though e-cigarettes are thought to be a more positive alternative to cigarettes, their chronic effects are still being investigated.

Bisphosphonates, either oral or intravenous, are an increasingly utilised medication in the treatment of osteoporosis in patients deemed at risk of fragility fractures. An anti-resorptive medication, bisphosphonates bind avidly to hydroxyapatite in the bone matrix. Here it is internalised by osteoclasts during bone resorption. Nitrogen present in the bisphosphonates inhibits farnesyl pyrophosphate synthase in the osteoclast’s mevalonate pathway, thereby impairing prenylation of small GTP-binding proteins, disrupting osteoclast function, and inducing apoptosis [106]. Bisphosphonates reduce the risk of major osteoporotic fractures by 33%, hip fractures by 33%, and vertebral fractures by 55% [107]. This manifests in the clinical guidelines; the National Institute for Health and Care Excellence (NICE) recommends the use of bisphosphonates in those in danger of OP after risk assessment, including secondary OP, and after initiating GCs or AIs [108,109]. Unfortunately, the use of bisphosphonates does not come without its side effects, and the development of atypical femoral fractures and osteonecrosis of the jaw are two that can be particularly unfavourable. Further guidelines advise initiating calcium and vitamin D alongside anti-resorptive medication [108].

Whilst bisphosphonates work by preventing bone resorption, an emerging pharmacological target is bone-forming anabolic agents. Teriparatide, abaloparatide, and romosozumab are currently used in severe OP secondary to menopause. Teriparatide and abalopratide are PTH analogues. These act on PTH1 receptors on osteoblasts and osteocytes. When given intermittently, they enhance osteoblast differentiation and survival, promoting bone remodelling toward formation. Romosozumab, by contrast, is a sclerostin inhibitor. By blocking sclerostin (an osteocyte-derived protein that suppresses the Wnt/β-catenin signalling pathway), it again stimulates osteoblast activity to inevitably favour bone formation whilst simultaneously suppressing resorption [110]. The Active-Controlled Fracture Study in Postmenopausal Women with Osteoporosis at High Risk (ARCH) trial compared treatment with alendronate alone versus that with romosozumab followed by alendronate. The study demonstrated that the combination therapy significantly reduced the risk of new vertebral fractures at 24 months (48%) and clinical fractures at 33 months (27%) [111]. NICE guidelines recommend the use of romosozumab in patients with severe post-menopausal OP who have had a major osteoporotic fracture in the preceding 24 months [112].

## Conclusions

Bone remodelling is a finely regulated process dependent on the balance between osteoblast-mediated bone formation and osteoclast-mediated bone resorption. This equilibrium is influenced not only by genetic and intrinsic factors but also by a wide array of environmental exposures. The evidence presented in this review highlights how modifiable environmental factors intersect with key molecular pathways such as RANK/RANKL/OPG to influence skeletal integrity. Importantly, these influences can be both detrimental and protective, underscoring the need for nuanced interpretation in clinical practice. Lifestyle interventions remain central to preserving bone health. Regular weight-bearing exercise enhances mechanotransduction through osteocytes and supports osteoblast differentiation, while smoking cessation and moderation of alcohol intake reduce inflammatory and oxidative stresses that favour osteoclastogenesis. Pharmacological interventions such as bisphosphonates provide an important adjunct in patients at heightened risk of fragility fractures. Their ability to disrupt osteoclast activity and reduce fracture incidence has established them as first-line therapy in both primary and secondary OP. In summary, safeguarding skeletal health requires a holistic, multidisciplinary, and preventative approach. By understanding how environmental and lifestyle factors modulate the biological drivers of bone remodelling, clinicians can adopt a more personalised strategy that integrates behavioural, nutritional, and pharmacological measures to mitigate OP and fracture risk across a patient’s life.
